# eDNAmap: A Metabarcoding Web Tool for Comparing Marine Biodiversity, With Special Reference to Teleost Fish

**DOI:** 10.1111/1755-0998.70066

**Published:** 2025-11-05

**Authors:** Jun Inoue, Junya Hirai, Kiriko Ikeba, Zeshu Yu, Sk Istiaque Ahmed, Zhen Lin, Yuan Lin, Marty Kwok‐Shing Wong, Chuya Shinzato, Sachihiko Itoh, Shin‐ichi Ito, Hiroaki Saito, Susumu Hyodo

**Affiliations:** ^1^ Atmosphere and Ocean Research Institute The University of Tokyo Kashiwa Japan

**Keywords:** biogeographic boundary, community comparisons, environmental DNA, map, teleost fish

## Abstract

Marine environmental DNA (eDNA) metabarcoding data are beginning to accumulate, even for remote and poorly studied areas, such as marine environments. These data enable us to identify distributions of target organisms and then to compare biological compositions between different marine areas. However, there is no platform to effectively utilise and accumulate these data. In this study, we developed eDNAmap, a web‐based platform designed to analyse and store marine eDNA metabarcoding data. By uploading species or sequence composition data with location information, eDNAmap users can automatically (1) plot sampling locations on a map, (2) generate a heatmap to evaluate potential batch effects arising from methodological differences and (3) perform nonmetric multidimensional scaling and cluster analyses using similarity indices. Furthermore, users can specify scientific names to display species distributions and upload species lists to assess species compositions of the target sea area. As an example, fish sequence composition data obtained from 55 stations around the Watase line—believed to exist along a geographic canyon known as the Tokara Gap—were used to verify its existence using eDNAmap. The platform includes a database primarily consisting of teleost fish data from the Northwestern Pacific, which users can analyse similarly to their own uploads. Although originally designed for fish, eDNAmap is flexible enough to handle data from other marine organisms. Analysing multiple taxa enables the detection of concordant biogeographic patterns across different groups, which can strengthen ecological interpretations and lay the groundwork for identifying environmental drivers shaping community structures. eDNAmap is available at https://github.com/jun‐inoue/eDNAmap.

## 1 Introduction

As environmental DNA (eDNA) metabarcoding data have begun to accumulate, it has become possible to understand distributions of marine organisms (Ruppert et al. 2019; Ye et al. 2025). This technology contributes to understanding biota and to conserving biodiversity in surveyed marine areas by creating feature tables consisting of species, operational taxonomic units (OTUs) or amplicon sequence variant (ASV) counts (Hakimzadeh et al. 2024). Particularly in the open ocean, where it is challenging to grasp species composition through organismal capture, eDNA data analysis is anticipated to be extremely useful. To support the effective use of these accumulating datasets, platforms are increasingly needed that can store, visualise and compare species‐composition data across diverse marine locations.

Existing databases that compile marine eDNA data are mostly limited to plankton, such as the Ocean Barcode Atlas (https://oba.mio.osupytheas.fr/ocean‐atlas/OBA_analyse; Vernette et al. 2021) and MetaZooGene (https://metazoogene.org/MZGdb; Bucklin et al. 2021). The Ocean Barcode Atlas compares compositions of species among user‐specified taxa, primarily in the Atlantic Ocean. MetaZooGene offers a reference database of genetic sequences and a barcode atlas. These databases, however, lack a web tool function for users to analyse their own species/sequence composition data. During the development of phyloBARCODER (Inoue et al. 2024), a tool for species identification for DNA metabarcodes, we recognised the need for a web tool that allows users to upload species composition data, map it, and compare it across marine areas.

**FIGURE 1:**
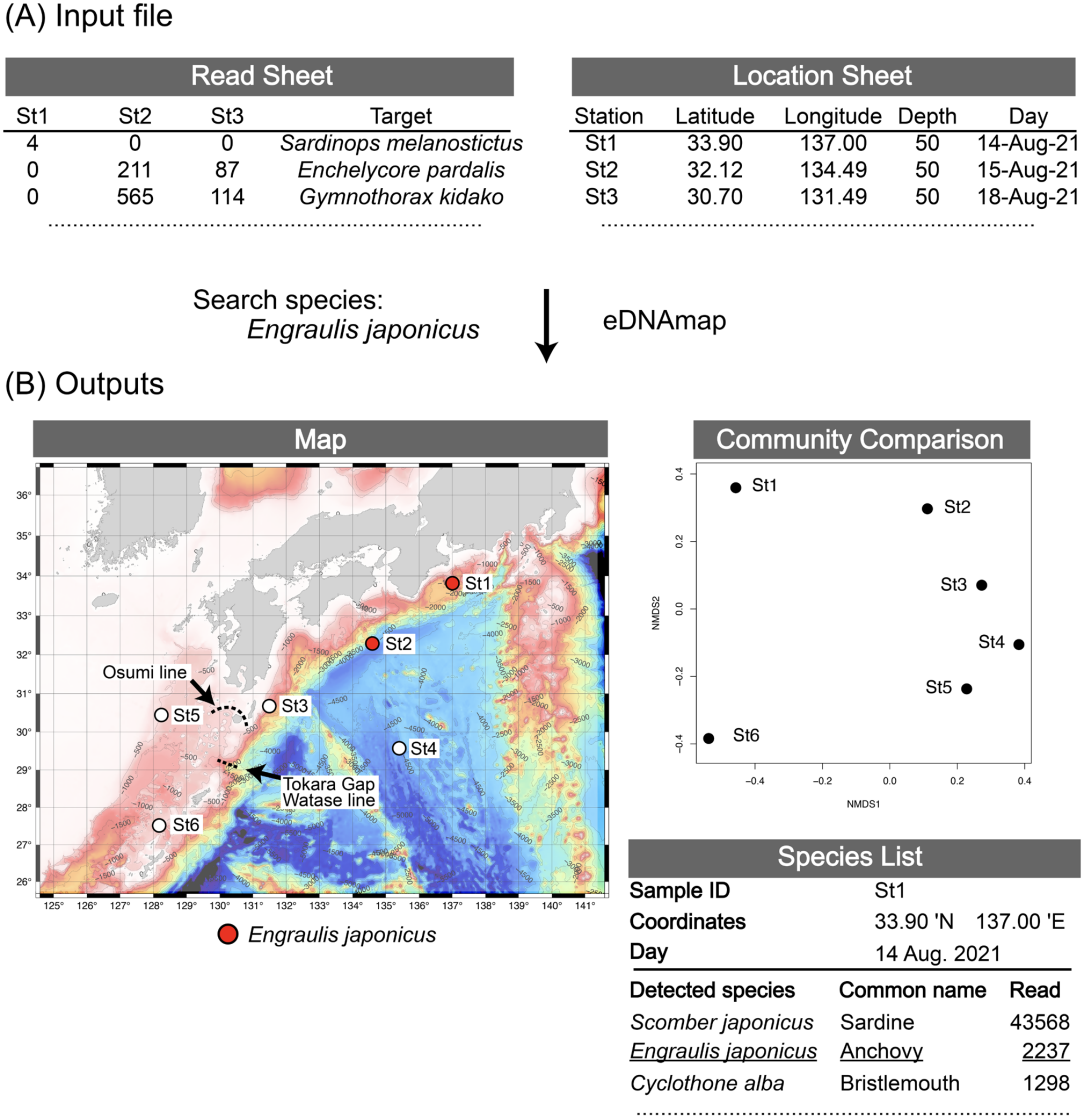
Overview of Ocean eDNAmap. (A) Input file. Users upload a single Excel file that combines the Read and Location Sheets. (B) Outputs. The Tokara Gap, a submarine canyon in the Ryukyu Archipelago (Motokawa and Kajihara 2017), and Watase (Komaki 2021) and Osumi (Motomura and Harazaki 2017) lines were added to the figure manually after downloading.

Comparing species compositions among marine areas using eDNA metabarcoding data may help verify biogeographical boundaries (Miya 2022; West et al. 2021). Around Japan, for both terrestrial and marine organisms, the Watase line has been believed to exist along a geographic canyon, the Tokara Gap (Figure 1) (Komaki 2021). However, the existence and location of this well‐known boundary have rarely been confirmed through comparisons of species composition. Recently, based on tests of seven hypothetical boundaries for terrestrial animals, Komaki (2021) analysed published species composition data and suggested that transition zones, rather than clearly defined boundaries, are more appropriate when describing biogeographic patterns. For marine organisms, although the more northerly Osumi line is proposed for fish (Motomura and Harazaki 2017), no verifiable data have been reported, other than population genetic data for two invertebrate species (Ogoh and Ohmiya 2005; Yorifuji et al. 2012). Accordingly, we collected eDNA metabarcoding data for teleost fish around the Tokara Gap in research cruises conducted in 2020 and 2022.

**FIGURE 2:**
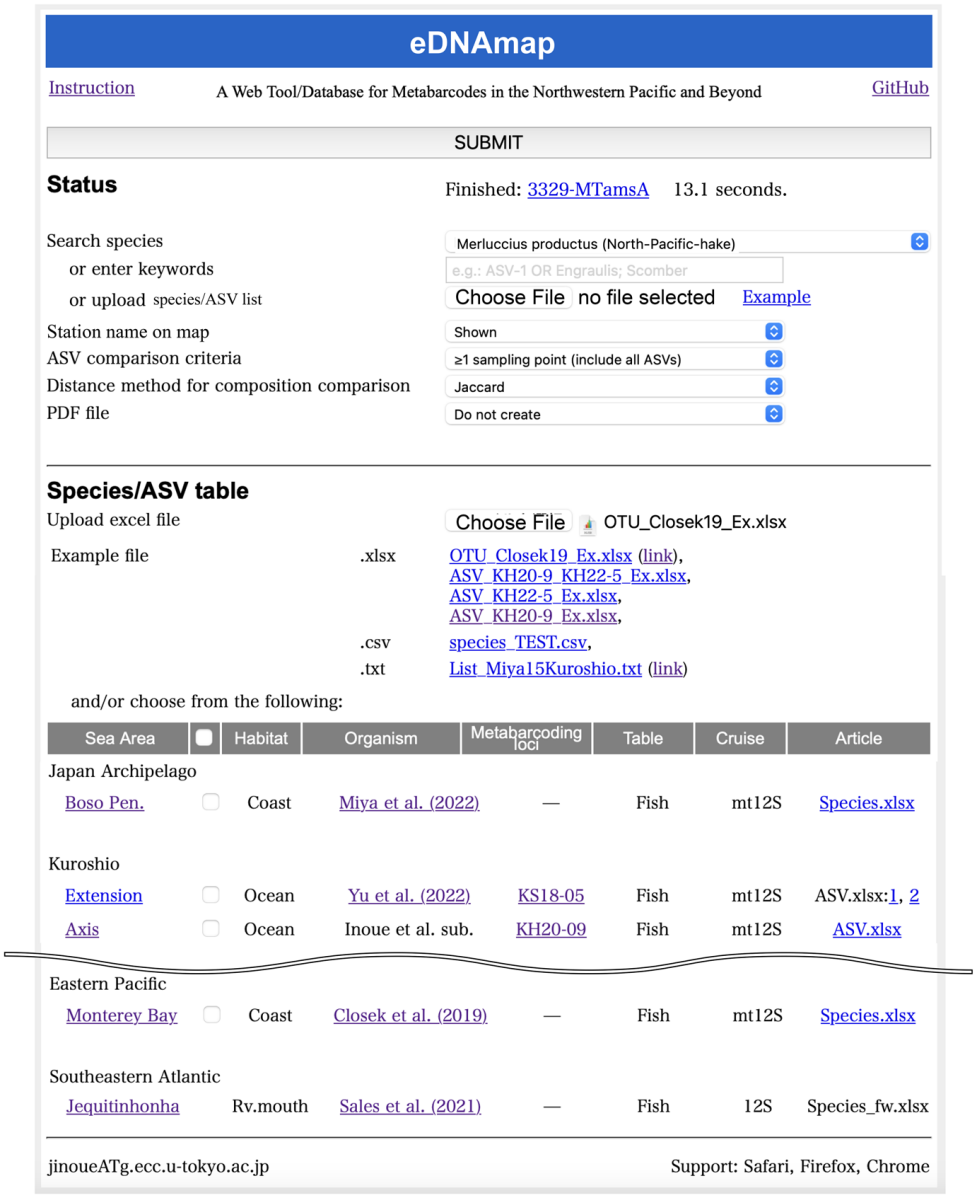
Top page of eDNAmap.

In this study, we developed a web tool and database called eDNAmap to analyse metabarcoding data, primarily targeting teleost fish. Users can upload species/ASVs composition lists for fish or other organisms and compare them across locations (Figure 1). In other words, eDNAmap supports consistent analysis of ASV tables derived from different genetic markers, enabling cross‐study comparisons. Before performing an analysis, users need to create a species/ASV table from raw fastq file data. eDNAmap allows users to compare sequence composition by ASV IDs without performing species identification. Since uploaded data include location data, sampling sites anywhere on the globe are automatically plotted on the map and locations where target species or ASVs are detected are highlighted. To illustrate this utility, we selected *Katsuwonus pelamis* —a widely distributed pelagic fish species with easily identifiable barcode sequences—as an example species. Those uploaded ASV tables are only accessible to the individual user and do not become part of the public database. Version 1 of the database consists of marine organismal data, primarily of teleost fish from the Northwestern Pacific, published in 12 papers, including three research cruises (Figure 2). To test whether eDNAmap can be used to verify biogeographic boundaries, we analysed data obtained from the Tokara Gap region.

## 2 Materials and Methods

### 2.1 Implementation

eDNAmap was created using Python, Python's CGI module and JavaScript. For map creation and plotting of water sampling stations, Generic Mapping Tools version 6.5.0 (Wessel et al. 2019) was used. To analyse Excel files consisting of species/ASV sheets and location sheets (Figure S1), the Python module pandas (The Pandas Development Team 2020) was utilised.

Analysis and visualisation of composition comparisons were performed using R packages (R Core Team 2020). Creation of heatmaps for species/ASV composition was done using pheatmap (Kolde 2019). Based on similarity indices, nonmetric multidimensional scaling (nMDS) and permutational multivariate analysis of variance (PERMANOVA) were performed using ‘metaMDS’ and ‘adonis’ functions, respectively, from the vegan package (Oksanen et al. 2019). Clustering was visualised using the function hclust.

The eDNAmap database consists of Excel files containing species/ASV tables created from metabarcoding sheets and location sheets (Figure S1A). Users can also upload their own Excel files for analysis, which are not added to our public database, even though these files are analysed together with the user‐selected data from this database. These user‐uploaded files remain private and are not accessible to other users. The dictionary that maps scientific names to common names was created using the R package, rfishbase (Boettiger et al. 2012) and EDirect, provided by NCBI (https://dataguide.nlm.nih.gov/edirect/documentation.html).

### 2.2 Sampling

**FIGURE 3:**
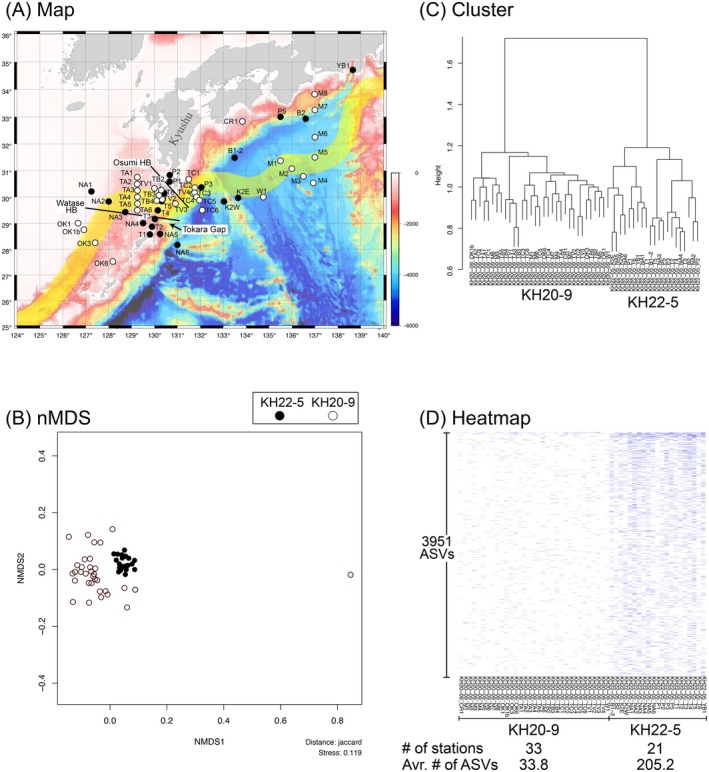
Outputs of eDNAmap. Teleost ASV compositions were compared among KH22‐5 and KH20‐9 stations. Raw data from both cruises were combined and preprocessed to create the ASV table. (A) Map. The Kuroshio was represented by a transparent yellow line, and the Watase and Osumi hypothetical boundaries (HBs) were added to the figure manually after downloading. (B) Results of nMDS analysis showing the similarity of ASV compositions. (C) Results of cluster analysis. (D) Heatmap showing the presence/absence of ASVs.

As an example of an analysis, data from the KH22‐5 and KH20‐9 cruises, collected around the Tokara Gap (Figure 1) and the Kuroshio region, were used. The Kuroshio is a western boundary current of the subtropical gyre in the North Pacific. It originates east of the Philippines, passes through the East China Sea west of Okinawa Island, and flows into the northern North Pacific through the Tokara Gap off Kyushu (Figure 3A).

Details of sampling methods are described in Wu et al. (2024) and Yu et al. (2022). Briefly, water was collected using Niskin bottles. Water sampling was conducted at 33 stations with a total of 131 bottles during the KH20‐9 cruise, and at 21 stations with 81 bottles during the KH22‐5 cruise. At each station, water was typically collected from up to four depth levels: 10 m, 50 m, 100 m and 150 m. However, the actual number of depths sampled at each station varied depending on local conditions and cruise design. Including six (KH22‐5) and one (KH20‐9) negative controls, 54 stations and 220 samples were used for the analysis (Table S1). eDNA was collected by filtering up to ~10 L of seawater through Sterivex‐GP cartridge filters (0.45 μm, Millipore SVHV010RS, Merck, Tokyo, Japan) using peristaltic pumps.

### 2.3 Sequence Generation

DNA extraction was performed in the laboratory according to Wong et al. (2020) and Yu et al. (2022). Sequencing was outsourced to Bioengineering Lab. Co. Ltd. Library preparation involved two‐step tailed PCR that targeted the 12S rRNA region with MiFish primers (Miya et al. 2015). After assessing the quality and concentration of the prepared libraries, sequencing was performed on NextSeq500 (KH20‐9) or HiSeq X (KH22‐5) systems under 2 × 150‐bp, paired‐end conditions. For preprocessing of data from fastq to fasta sequences, an analytical pipeline, Qiime2 version 2024.10.1 (Bolyen et al. 2019) was used with the amplicon sequence variant (ASV) method implemented in the DADA2 package (Callahan et al. 2016). Primers were removed and filtered using cutadapt (Martin 2011). Then, sequences were de‐replicated, error‐corrected, and merged to produce an ASV‐sample matrix. Chimeric sequences were removed. All singletons, doubletons and tripletons per sample were removed from subsequent analyses to avoid false positives (Edgar 2010).

### 2.4 Species Identification

Species identification was primarily performed using BLAST searches against the MIDORI2 long database (Leray et al. 2022), conducted independently of the web tool. This database consists of nucleotide sequences of the mitochondrial 12S rRNA gene, representing 57,969 sequences of various metazoan species registered in NCBI. Based on the top three species names obtained from BLAST search results, nonteleost ASVs and freshwater fish ASVs identified by rfishbase were removed. Additionally, ASVs detected in the negative controls were excluded, resulting in a final total of 4847 ASVs.

For database registration in eDNAmap, species identification was performed solely using similarity analysis and was not confirmed through phylogenetic tree estimation (Czech et al. 2022). Given the large number of obtained ASVs and the limited teleost reference database for the open ocean (Miya 2022), it is impractical to assign species names to all ASVs through phylogenetic tree identification.

However, in the analysis of visualising species distribution, species identification of the corresponding ASV sequences was confirmed by estimating a phylogenetic tree using phyloBARCODER (Inoue et al. 2024). As an example, we selected *K. pelamis* , which has a broad distribution and belongs to a monotypic genus (Nelson et al. 2016); these characteristics increase the likelihood of accurately assigning ASVs to the species based on phylogenetic trees.

For calculation of community similarity indices of KH22‐5 and KH20‐9 teleost data, ASV IDs were used instead of identified species names. Community similarity indices are expected to be calculated more accurately using ASV IDs than OTU IDs (Callahan et al. 2017) or species names, due to their ability to resolve single‐nucleotide differences and retain fine‐scale biological variation (Chiarello et al. 2022).

## 3 Results

### 3.1 Interface

Figure 2 shows the top page of eDNAmap version 1. Currently, eDNAmap primarily targets teleost fish from the open ocean in the Northwestern Pacific. However, for comparison, it also includes teleost fish data from coastal waters of Boso Peninsula, Honshu, Japan (Miya et al. 2022), Palmyra Atoll (Lafferty et al. 2021) and the Belgian coast of the North Sea (Dukan et al. 2024). In addition to teleost fish, the database also includes data on dinoflagellates (Wu et al. 2024) and corals (Nishitsuji et al. 2023; Shinzato et al. 2021), which are expected to accumulate in the future. Each dataset consists of a single Excel file, comprising species/ASV and location sheets (Figure S1).

For eDNAmap analyses, users can upload their own Excel files (Figure S1A) or select buttons from the list to use pre‐installed ASV tables. eDNAmap also supports integrated analyses combining user‐uploaded ASV files with pre‐installed ones. These pre‐installed ASV tables can be downloaded from the top page of eDNAmap. To demonstrate that locations outside the Northwestern Pacific can also be plotted, we used OTU_Closek19_Ex.xlsx, which contains a species table obtained from the Monterey Bay area (Closek et al. 2019). This file can be downloaded from the right side of the Example section on the eDNAmap top page (Figure 2). When this Excel file is uploaded and analysed, the process completes in ~14 s, and output is generated (Figure S2). To verify whether the numbers of reads in ASV tables are sufficiently amplified, the user is recommended to evaluate the rarefaction curve by depicting functions such as the rarecurve function implemented in the vegan package in R, before uploading ASV tables for eDNAmap analyses.

The ‘Search species’ option (Figure 2) highlights sampling stations on the output map at which specified species or ASV IDs were detected. The user‐defined ‘ASV comparison criteria’ option excludes rarely detected species/ASVs from nMDS and cluster analyses by specifying the required minimum number of sampling points at which each ASV must be detected. The ‘Distance method for composition comparison’ option defines the Jaccard distance (converging to presence/absence data) or the Bray‐Curtis distance (taking read counts into account) for calculating community similarities between stations/points. When uploading data, users need to pre‐exclude unnecessary data such as ASV sequences derived from negative controls.

Summary output (Figure S2) can be accessed by clicking the link created after the top page ‘Finished’. This summary output starts with a ‘Sampling site map’ and ‘Detection depth’ showing distributions of ASVs. ASV composition is depicted as a ‘pheatmap’. After that, ‘nMDS’ and ‘Cluster’ compare community composition among sampling points. Heatmap, nMDS and cluster analyses can also be visualised as ‘Sampling stations’, if the user has described multiple sampling points, for example, depths, at the same geographic location (Figure S1A). After that, the output shows a ‘Species list’. This list includes assigned species names and corresponding read counts detected at each sampling point. Then output ends with ‘Dependencies’, listing software used. Users can download output from the ‘Download’ link at the top of this summary.

### 3.2 Case Studies

To demonstrate the use of eDNAmap, we utilise teleost fish data obtained from two research cruises, KH22‐5 and KH20‐9. In addition, as an example of data analysis for organisms other than teleost fish, we use the ASV table derived from dinoflagellate metabarcoding data obtained from KH20‐9 (Wu et al. 2024).

### 3.3 Case Study 1: Verification of Analytical Biases

Case Study 1 illustrates that eDNAmap assesses potential biases, including batch effects, which may arise from integrating data across different projects. As an example, we used an ASV table created by preprocessing fastq data combined from the KH20‐9 and KH22‐5 cruises. This ASV table can be downloaded from the right side of the Example section on the eDNAmap top page (Figure 2). We conducted this analysis with default settings, using a threshold of ≥ 1 for ASV comparison criteria to include all detected ASVs. For calculating community similarities between stations/points, the Jaccard index, which emphasises presence/absence data, is used. This approach is particularly suitable for fish eDNA, where detection is strongly influenced by behavioural factors (dos Santos and Blabolil 2025).

Given that the raw data from the KH22‐5 and KH20‐9 cruises were processed using distinct sequencing platforms, we anticipated that the resulting ASV table would be affected by batch effects from the outset. Nevertheless, we initially hypothesised that geographically close sampling stations (Figure 3A), even from different cruises, would have similar ASV compositions, assuming that biological signals would outweigh technical biases. However, in the nMDS plot (Figure 3B), the sampling points from KH22‐5 clustered in the center, with the KH20‐9 sampling points distributed around them. The cluster analysis (Figure 3C) also showed that samples from KH22‐5 and KH20‐9 formed distinct clades. Upon examining the heatmap produced by eDNAmap (Figure 3D), we found that the average number of ASVs detected per sampling station was 205.2 for KH22‐5, compared to only 33.8 for KH20‐9.

We concluded that this difference in the number of ASVs detected per cruise hindered the estimation of biological similarities among sampling stations. To account for methodological differences that affected ASV detection, we decided to analyse samples from the KH22‐5 and KH20‐9 cruises separately by creating two ASV tables from raw data collected during each voyage.

### 3.4 Case Study 2: Comparison of ASV Compositions

Case Study 2 demonstrates that eDNAmap can be used to verify biogeographic boundaries. For this verification, we extended already proposed biological boundaries to the open ocean, setting the Watase (Komaki 2021) and Osumi (Motomura and Harazaki 2017) hypothetical boundaries (HBs) (Figure 3A). Colour coding of the nMDS plot and the PERMANOVA analysis was performed using R scripts included in the downloadable file (Figure S1B). Excel files containing ASV compositions for KH22‐5 and KH20‐9 can be downloaded from the top page.

**FIGURE 4:**
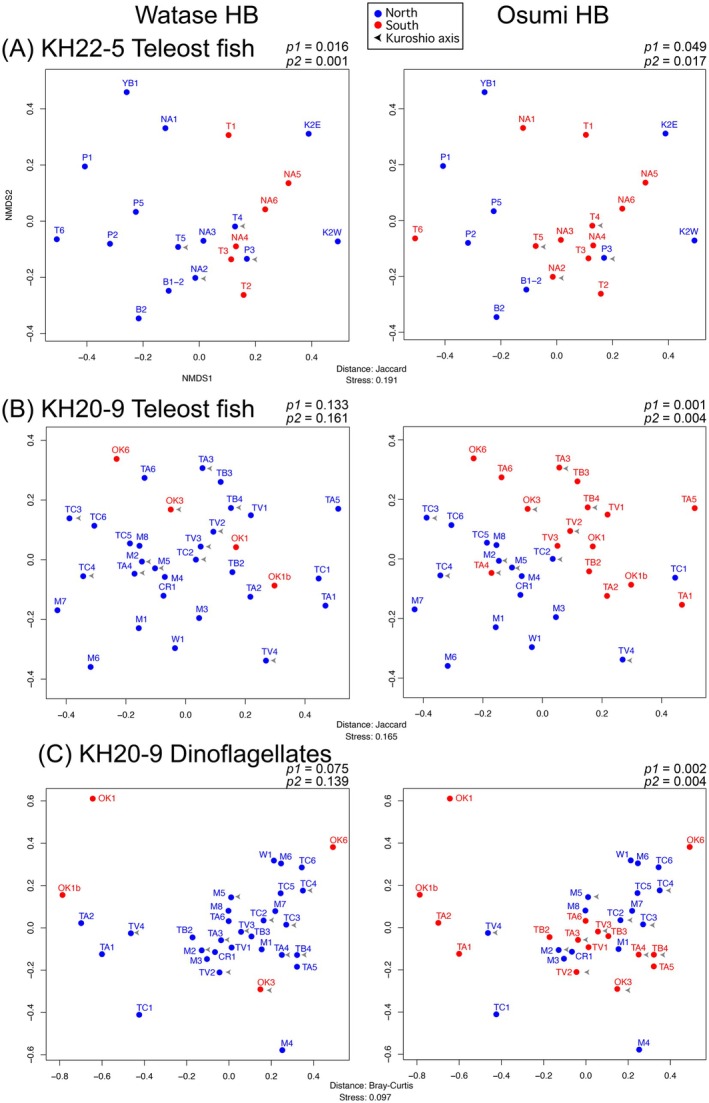
Results of nMDS analysis showing the similarity of ASV compositions detected in (A) KH22‐5 teleost fish, (B) KH20‐9 teleost fish and (C) KH20‐9 dinoflagellate data. Sampling stations located to the north or south of the Watase and Osumi hypothetical boundaries (HBs) were colour‐coded to distinguish regional differences. *p*‐values were calculated using PERMANOVA with the R script included in the output. *p*1 indicates values calculated by including stations located in the Kuroshio axis (arrowheads), while *p*2 indicates values calculated without them. The path and range of the Kuroshio Current for KH22‐5 follows the Hydrographic and Oceanographic Department, Japan Coast Guard (https://www1.kaiho.mlit.go.jp/jhd‐E.html), while KH20‐9 follows Wu et al. (2024).

In KH22‐5, metabarcode compositions of fish among stations were compared by nMDS analysis (Figure 4A). The consistent composition of southern communities across both Watase and Osumi HBs, especially in the Osumi region, suggests a biogeographic distinction between southern and northern regions. Differences were supported at the 5% significance level (*p1* = 0.016 and *p1* = 0.049 respectively). Similarly, in KH20‐9, division by the Osumi HB was statistically supported (*p1* = 0.001) (Figure 4B). However, no significant difference at the 5% level was detected on either side of Watase HB (*p1* = 0.133), and this value also does not indicate significance at the 10% level, suggesting that the observed differences are not statistically supported.

To account for transport of fish by the Kuroshio current, data were analysed using PERMANOVA after excluding stations located in the Kuroshio axis (Figure 4). In KH22‐5 (Figure 4A), this analysis indicated stronger statistically significant differences in both Watase (*p2* = 0.001) and Osumi HB (*p2* = 0.017). However, in KH20‐9 (Figure 4B), after excluding such points, statistically significant values did not become stronger (Watase: *p2* = 0.161; Osumi: *p2* = 0.004).

### 3.5 Case Study 3: Comparison Between Taxa

Case Study 3 demonstrates that eDNAmap can analyse eDNA data from organisms other than fish. Wu et al. (2024) analysed the 18S rRNA V4 region of metabarcoding data from dinoflagellates obtained from environmental water during the KH20‐9 cruise. From the top page (Figure 2), we checked the button for the dinoflagellate species table incorporated into the database (sheet 3 in Wu et al. 2024) and performed the eDNAmap analysis. We selected the Bray‐Curtis method, following Wu et al. (2024), as it better captures quantitative differences in sequence abundance for ubiquitously distributed organisms like dinoflagellates (dos Santos and Blabolil 2025).

Those results showed that there was a statistically supported difference in species composition on the two sides of the Osumi HB for dinoflagellates (Figure 4C) (*p1* = 0.002), but not for the Watase HB (*p1* = 0.075). After excluding points on the Kuroshio axis, statistically significant values did not become stronger (Watase: *p2* = 0.139; Osumi: *p2* = 0.004). Distributions of dinoflagellates may be influenced by properties of the water (Wu et al. 2024), rather than just by their location inside or outside the Kuroshio axis.

eDNAmap also compares species composition by depth. When the fish ASV tables of KH22‐5 and KH20‐9 were subjected to eDNAmap analysis with default settings, the nMDS plot for the KH20‐9 data (Figure S3A) showed that sampling points with low ASV detection frequencies were scattered, whereas most points with high detection frequencies overlapped at the same position. Therefore, for the KH20‐9 data analysis, we set the ASV comparison criteria to ‘≥ 5 sampling points’ to exclude ASVs with low frequencies. As a result (Figure S3B,C), no differences by depth were observed in teleost fish community compositions in the study area of KH20‐9 (*p* = 0.826), as were shown in KH22‐5 (*p* = 0.291) with default settings. In contrast to teleost fish, Wu et al. (2024) reported that dinoflagellate communities in the Kuroshio region were broadly divided into three groups: the surface layer (10 and 50 m), the lower part of the euphotic zone (100 m) and depths below the euphotic zone (150 m).

For a deeper investigation into the correlation between clearly divided community structure and environmental factors, eDNAmap provides, as output, an R script including the envfit function of R, which was used to create Figure S6. Detailed analyses were not performed, and the results were not discussed, as environmental factors fall outside the main scope of eDNAmap version 1.

### 3.6 Case Study 4: Matching Species Lists

Case Study 4 demonstrates that eDNAmap can compare uploaded plain text listing species names with those stored in the database. As an example of its use in artificial environments—such as aquariums rather than natural ecosystems—we compared the species list of fish collected in a Kuroshio tank of Okinawa Churaumi Aquarium (Miya et al. 2015) with those created in the Kuroshio region during the KH22‐5 and KH20‐9 cruises. The Kuroshio tank predominantly keeps large fish characteristic of areas around the Kuroshio.

We uploaded the List‐Kuroshio.txt file downloaded from the top page of eDNAmap (Figure 2) and checked the KH22‐5 and KH20‐9 buttons for analysis. Of the 44 species in the Kuroshio tank, four were detected in KH22‐5 (Figure S4A) and three in KH20‐9 (Figure S4B). Of these, *Caranx ignobilis* and *Katsuwonus pelamis* were commonly detected in both cruises, with the latter species being among the top 20 frequently detected species in KH22‐5 (Table S2).

These results suggest that many species displayed in the Kuroshio tank do not widely and consistently occur in the vicinity of the Kuroshio Current. Since assigned species can vary depending on the database and identification methods, for more detailed comparisons, common ASVs need to be rigorously determined by preprocessing combined raw data.

### 3.7 Case Study 5: Species/ASV Distribution

Case Study 5 demonstrates that eDNAmap can display the geographical distribution of species or sequences by providing keywords. From the Search species option (Figure 2), we selected *Katsuwonus pelamis* , which is distributed from the tropical to the temperate Pacific (Mugo et al. 2020), and chose five projects conducted in the Pacific for eDNAmap analysis.

**FIGURE 5:**
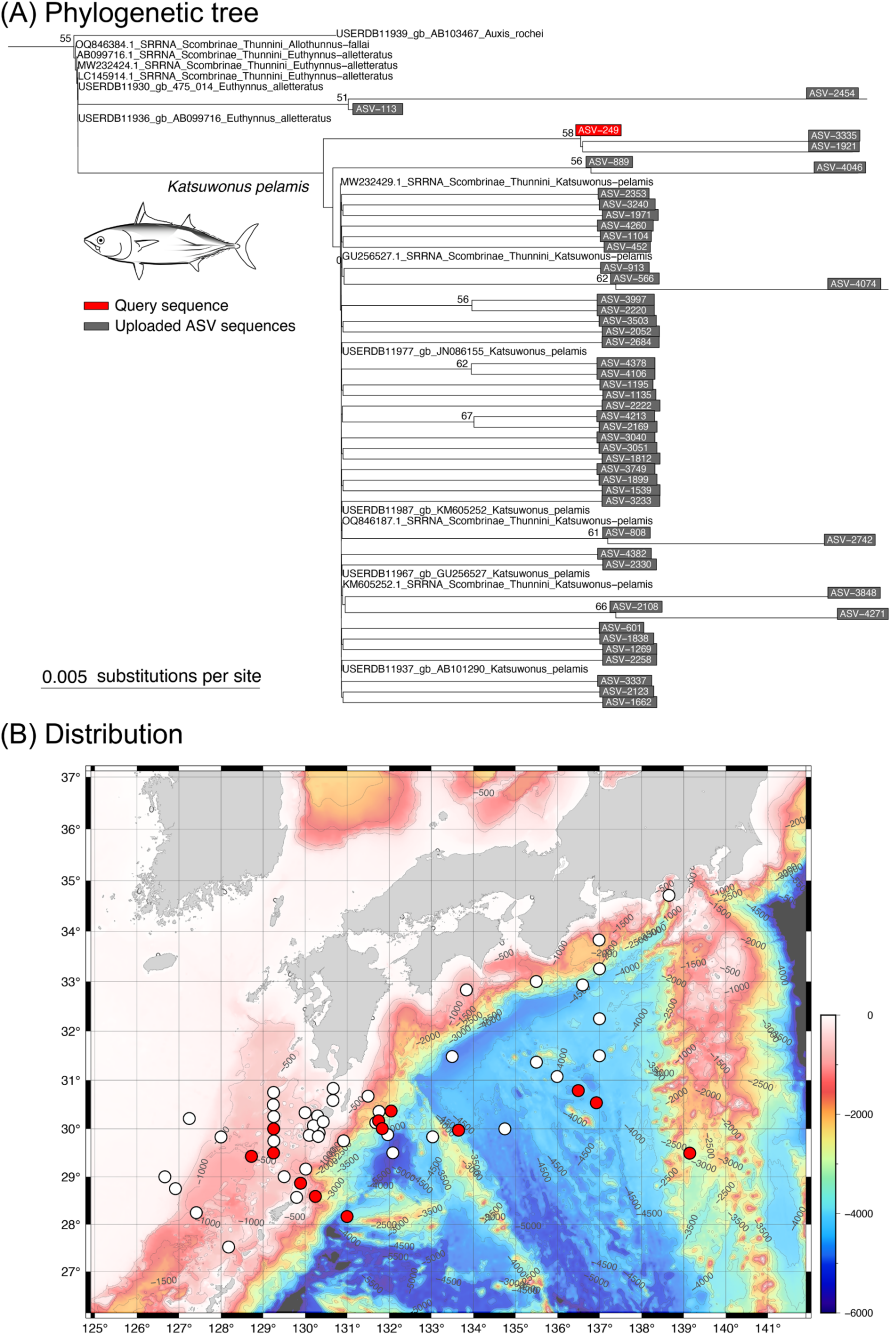
Phylogenetic tree (A) and distribution (B) of *Katsuwonus pelamis* ASVs detected in the KH22‐5 and KH20‐9 cruises. The phylogenetic tree was created using phyloBARCODER. Numbers near branches indicate bootstrap values. White circles indicate stations where *Katsuwonus pelamis* was not detected, while red circles indicate stations where *K. pelamis* was detected.

Among 67 stations, *K. pelamis* was detected at 17, including Palmyra Atoll (Lafferty et al. 2021) (Figure S5). To investigate its distribution in waters around Japan, we delineated ASV sequences of *K. pelamis* from the KH22‐5 and KH20‐9 ASVs by estimating the phylogenetic tree (Figure 5A) using phyloBARCODER (Inoue et al. 2024) with the MIDORI2 database.

We then uploaded the list of selected 47 ASV‐IDs (.txt file) along with the ASV table (.xlsx file) from the eDNAmap top page. As a result (Figure 5B), *K. pelamis* ASVs were distributed among 13 southern stations in the survey area. In addition, almost all ASVs were detected at depths of 10 or 50 m (Table S3), consistent with their known habitat in the upper mixed layer (Mugo et al. 2020).

## 4 Discussion

eDNAmap provides a versatile platform for exploring marine biodiversity patterns, including analytical biases, biogeographic boundaries and species distributions.

### 4.1 Analytical Biases in Jointly Generated ASV Tables

When combining eDNA data from multiple cruises, analytical biases such as batch effects can obscure biological signals and complicate community‐level comparisons. The heatmap generated by the uploaded ASV table (Case Study 1) showed significant differences in the number of species and reads between the KH22‐5 and KH20‐9 cruises (Figure 3D). Here, the ASV table was generated by jointly preprocessing the raw data obtained from the two cruises. While observed differences in numbers may partly reflect true ecological variation, such as seasonal changes, the large discrepancy strongly suggests that batch effects—most likely due to differences in sequencing platforms—were the primary source of bias.

This discrepancy may also reflect other possible sources of bias in eDNA‐based analyses, including contamination, PCR artefacts or sequencing errors (Deiner et al. 2017). These issues can lead to false positives or incorrect ASV assignments, although in this study, reads from negative controls were removed and error‐correction pipelines such as DADA2 were applied. Variations in library preparation may also have affected the number of detected species and read counts. Additionally, the transport of eDNA by marine currents can obscure spatial signals (Miya 2022), resulting in the detection of DNA from organisms that are not locally present.

### 4.2 Validation of Biogeographic Boundaries

This study demonstrates that eDNAmap can be effectively used for biogeographical analysis, at least in the Tokara Gap region. For example, eDNAmap compared community compositions of fish and dinoflagellates between Watase and Osumi hypothetical boundaries (Figure 4). Such boundary positioning is not necessarily definitive, as results depend on the ASV tables used and the spatial resolution of sampling. In addition, for highly mobile taxa such as fish, it may be difficult to precisely delineate boundaries based solely on species compositions. Similarly, for widely distributed taxa like dinoflagellates, environmental factors may obscure clear boundaries. Furthermore, Costello et al. (2017) suggested that biogeographic boundaries should be defined not by species composition alone, but by endemicity, which better reflects evolutionary history and regional uniqueness. Nevertheless, eDNAmap serves as a useful starting point for evaluating boundary hypotheses.

Results from both KH22‐5 and KH20‐9 cruises (Figure 4) suggested that species compositions differed between the northern and southern sides of Watase or Osumi HB, although some comparisons did not yield statistically significant values. Biogeographic differentiation observed across both boundaries suggests that, around the Tokara Gap, the biogeographic boundary in the open ocean is band‐like rather than line‐like, as suggested for terrestrial boundaries (Komaki 2021).

Among the two hypothetical boundaries, statistically significant values were generally stronger for the Osumi HB than for the Watase HB (Figure 4). One possible reason is the greater number of sampling stations on the south side of the Osumi HB, which may have increased statistical support. To more clearly evaluate the boundary, additional sampling stations—especially on the southern side of the Watase HB—were needed.

### 4.3 Comparison With Other Software Programs

To the best of our knowledge, no web tool allowed users to upload species/ASV lists obtained as eDNA metabarcoding data to analyse among stations.

eDNAmap allows users to upload and analyse their own ASV tables (Case Studies 2 and 3). Researchers can conduct customised biodiversity analyses using unpublished or region‐specific data. Recently, the importance of eDNA metabarcoding data has been recognised, leading to the development of the ASV Registry (Bräunig et al. 2024), which stores and curates species/ASV tables. By downloading the ASV table provided by the ASV Registry and using it as an input file for eDNAmap, users can plot sample locations and compare species/ASV compositions without the burdensome task of preprocessing raw data.

eDNAmap also supports integration of user‐uploaded ASV tables with pre‐installed ones, facilitating comparative studies across different regions. This flexibility makes eDNAmap a powerful tool for expanding biodiversity research beyond the Northwestern Pacific, including other oceanic regions and taxonomic groups. Furthermore, eDNAmap can display the geographical distribution of species or sequences (Case Study 5), enabling researchers to integrate their findings with broader themes such as species distribution and detection patterns.

### 4.4 Potential Applications

Although case studies were for teleost fish and dinoflagellates, eDNAmap is applicable to a wide range of marine organisms, including corals and plankton, and potentially to terrestrial ecosystems beyond the marine realm. In addition to its core functionalities such as species boundary delimitation, eDNAmap can be used for monitoring biodiversity over time, identifying range shifts, or detecting invasive species. Moreover, eDNAmap enables users to compare independently compiled species lists—such as those from biodiversity inventories—with the eDNAmap database, as shown by the Kuroshio tank list (Case Study 4). Additionally, users can employ the provided scripts (Figure S6) to explore which environmental variables most significantly influence species composition similarities across sites.

### 4.5 Future Development

In the long term, eDNAmap may also support direct input of fastq files and automatically generate ASV tables with species names assigned using reference databases. However, due to the high computational load and long processing times required, version 1 of eDNAmap does not implement those functions.

For users interested in exploring environmental influences, an R script to facilitate such analyses is provided (Figure S6). Although we recognise that such environmental parameters can support standardised comparisons, we do not plan to incorporate more detailed parameters into the ASV tables used in eDNAmap's automatic analysis. This is because types of environmental parameters are highly variable, and even basic parameters such as temperature and salinity are not consistently reported in original publications (Shea et al. 2023).

### 4.6 Caveats

(i) At the time of the release of version 1, available ASV table data are limited in both taxonomic and geographic scope, and mostly derived from 12S rRNA regions; it is necessary to enrich the database with data from other genetic markers and marine regions through future publications and research cruises. (ii) When strictly comparing species/ASV tables derived from different cruises or DNA library protocols, it is necessary to create ASV tables by combining fastq data. (iii) When comparing ASV tables in the database, users should be aware of potential biases arising from methodological differences, including sampling design, primer selection, laboratory protocols, sequencing platforms, sequencing depth and bioinformatics pipelines (Deiner et al. 2017).

## Author Contributions

J.I. and S.H. conceptualised the workflow of eDNAmap. J.I. developed the software. J.I., J.H. and Z.Y. tested the program. J.I., K.I. and S.H. set up and assembled sampling equipment for the project. J.I., Z.Y., S.I.A., Z.L., and C.S. conducted water sampling, and H.S. designed research cruises. M.K.‐S.W. extracted DNA. J.I. and J.H. prepared the earliest version of the manuscript. J.I., J.H., Z.Y., S.I.A., Z.L., Y.L., M.K.‐S.W., C.S., S.I., S.‐I.I. and H.S. edited the manuscript.

## Disclosure

Benefits Generated: This research supports biodiversity monitoring by sharing eDNA data and the eDNAmap tool on public platforms, promoting open science and capacity building.

## Ethics Statement

The authors have nothing to report.

## Conflicts of Interest

The authors declare no conflicts of interest.

## Supporting information


**Table S1:** Correspondence table for SRR ID, sample ID and station.

**Table S2:** Number of detected sampling points for species in the Kuroshio list (Miya et al. 2015).

**Table S3:** Depth‐specific detection frequencies of *Katsuwonus pelamis* ASV obtained from the KH22‐5 and KH20‐9 cruises.

**Data S1:** Supplementary Figures.

## Data Availability

Raw read data have been submitted to NCBI (BioProject: PRJNA1241902; BioSample Accession nos.: SAMN47574851–SAMN47575070; SRA accessions: SRR32859190–SRR32859409). Sequenced reads have been deposited at Zenodo (10.5281/zenodo.15229240). The source code is available for review and reproducibility on the first author's GitHub repository. Output from eDNAmap may be used for commercial purposes, but its script may not be modified or uploaded on a web server without permission.
