# Supplementary material for: eDNAmap: A Metabarcoding Web Tool for Comparing Marine Biodiversity, With Special Reference to Teleost Fish
Source: Mol Ecol Resour. 2025 Nov 5;26(1):e70066. doi: 10.1111/1755-0998.70066 (PMC12627913; doi:10.1111/1755-0998.70066)
Supplement: Supplementary file 4 — Data S1: Supplementary Figures. [file MEN-26-e70066-s003.pdf]

MOLECULAR ECOLOGY RESOURCES

Supplemental Information for:

eDNAmap: A Metabarcoding Web Tool for Comparing Marine Biodiversity, with Special Reference to Teleost Fish

Inoue et al.

Table S1 | Correspondence table for SRR ID, sample ID, and station.

Table S2 | Number of detected sampling points for species in the Kuroshio list (Miya et al. 2015).

Table S3 | Depth-specific detection frequencies of *Katsuwonus pelamis* ASV obtained from the KH22-5 and KH20-9 cruises.

FIGURE S1 | Examples of input and output files for eDNAmap. (A) Input file. Two sheets can be uploaded as one Excel file. (B) Output file.

FIGURE S2 | Output file of eDNAmap.

FIGURE S3 | Comparison of ASV composition among sampling points at different depths. (A) KH20-9. All ASVs were used. The red arrow indicates the point at which most plots overlap. (B) KH22-5. (C) KH20-9. Using the ASV comparison criteria option, only ASVs detected at ≥ 5 sampling points were used.

FIGURE S4 | Comparison between ASV detection and the uploaded species list. (A) KH22-5. (B) KH20-9. Species names present in both the Kuroshio list (Miya et al. 2015) and the KH22/KH20-9 lists are indicated with red text and lines.

FIGURE S5 | Map plots of the eDNAmap species search. This plot was created by choosing five species/ASV tables from the eDNAmap database: KH22-5 and KS18-5 from Kuroshio Current, Kim et al. (2022) from Tsushima Current, Miya et al. (2022) from Boso Peninsula, and Oka et al. (2020) from Okinawa Island. Red plots indicate stations at which *Katsuwonus pelamis* was detected.

FIGURE S6 | An example of fitting analysis between community structure and environmental factors. By using output files of eDNAmap (620_nMDS.R, 200_communityData4R.csv, 200_environmentData4R.csv.), this analysis was done with the envfit function of R. To obtain eDNAmap outputs, an analysis was conducted with the ASV comparison criteria of ≥ 5 and the distance method of Jaccard using the downloaded ASV_KH20-9_Ex.txt file from the top page.

(A) Input file

Read sheet

Samp1	Samp2	Samp3	Samp4	Samp5	Samp6	Target
0	211	87	0	0	0	Enchelycore pardalis
0	565	114	0	150	0	Gymnothorax kidako
0	0	0	0	8	0	Gymnothorax reticularis
0	0	0	0	24	0	Xyrias sp.

Location sheet

SampleID	Cruise	Station	Latitude	Longitude	Depth	Day
Samp1	Test	St1	45.83	155.99	50	18-Aug-21
Samp2	Test	St1	45.83	155.99	100	14-Aug-21
Samp3	Test	St2	27.52	128.18	50	22-Jul-21
Samp4	Test	St2	27.52	128.18	100	12-Aug-21
Samp5	Test	St3	30.67	131.49	50	26-Jul-21
Samp6	Test	St3	30.67	131.49	100	21-Aug-21

(B) Output files

000_Species_Closek19_Ex.xlsx	Excel file including 2 sheets in (A).
010_results.html	Summary
150_map.png	Map
200_communityData4R_depth.csv	ASV table for depth detection.
200_communityData4R_station.csv	ASV table for station detection.
200_communityData4R.csv	ASV table for point detection.
200_environmentData4R.csv	
210_pheatmap_depth.png	Heatmap of ASV compositions
210_pheatmap_station.png	
210_pheatmap.png	
220_nMDS_station.png	nMDS plot.
220_nMDS.png	
230_hclust_station.png	Cluster.
230_hclust.png	
610_pheatmap.R	R scripts for downstream analyses.
620_nMDS.R	
625_permanova.R	
630_hclust.R	

FIGURE S1

eDNAmap
(ver.1.0.0)

Download:result2979_oeDNAmap.zip

### Setting

Cruise/Research : OTU_Closek19_Ex.xlsx

Search species : Merluccius-productus (North-Pacific-hake) FishBase

---

### Sampling site map

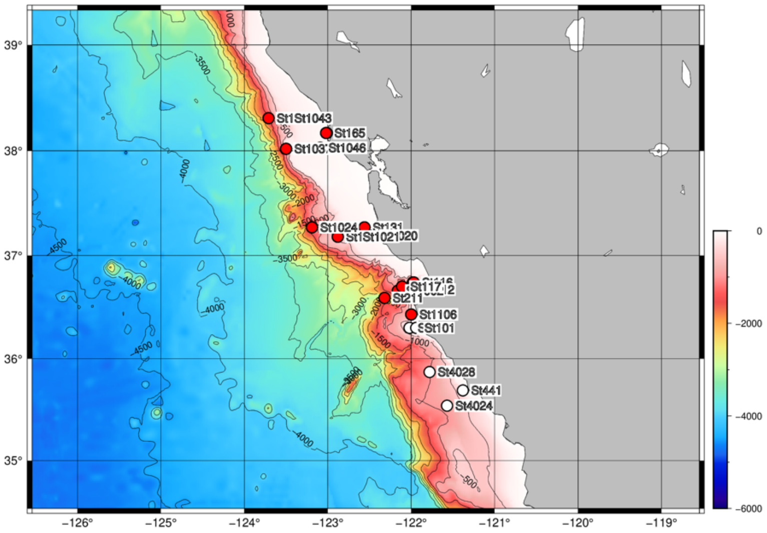

Red sites: Merluccius-productus (North-Pacific-hake) were detected.

### Detection depth

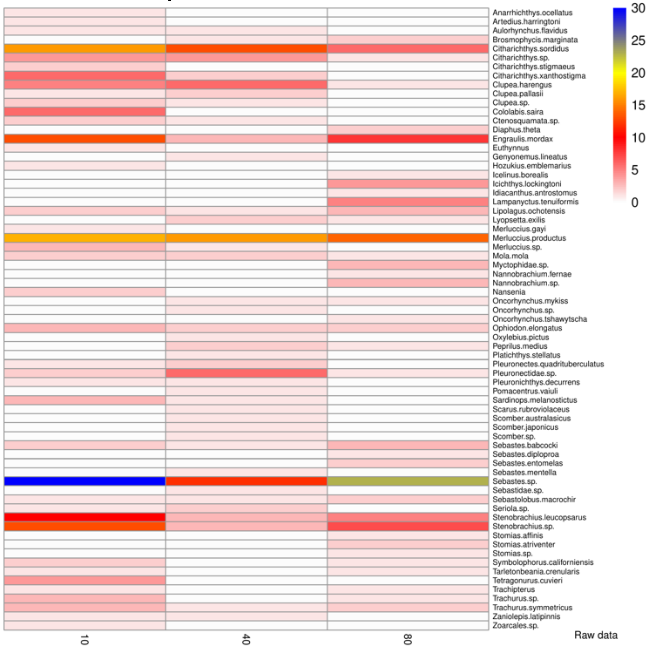

Color-coded by detection frequency.

FIGURE S2–1

Comparing Species/ASV composition

**Community data**	: User-xlsx
**ASV comparison criteria**	: ≥ 1 sampling points
**Removed sampling point**	: NONE
**200_communityData4R.csv**	: 131 points, 87 spp.
**200_communityData4R_station.csv**	: 21 stations, 87 spp.

Caution:
When comparing ASVs across different projects, users should consider potential biases due to methodological differences, such as sampling design, primer selection, laboratory protocols, sequencing platforms, and bioinformatics pipelines (Deiner et al. 2017; Shea et al. 2023).

pheatmap

Sampling point

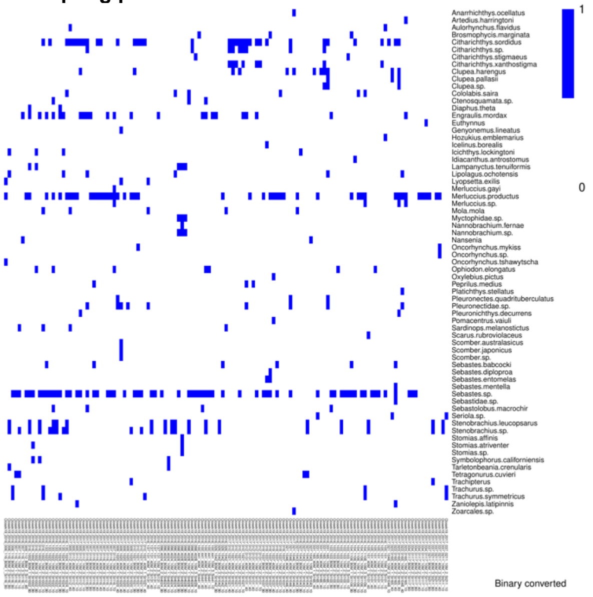

Sampling station

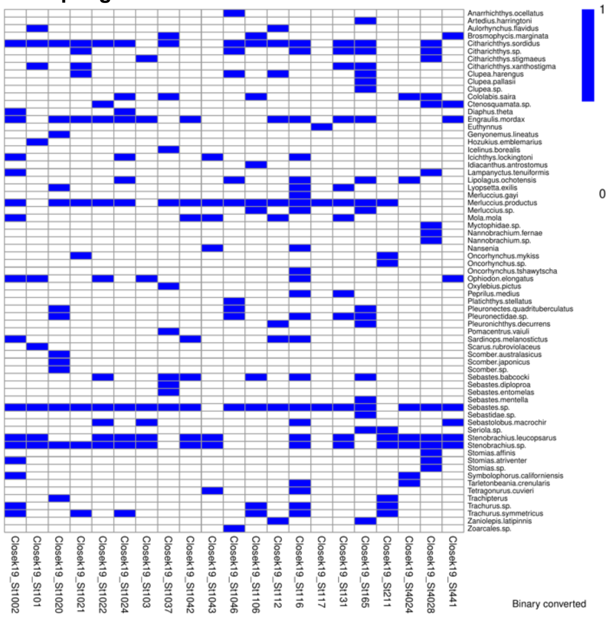

FIGURE S2–2

nMDS

Sampling point

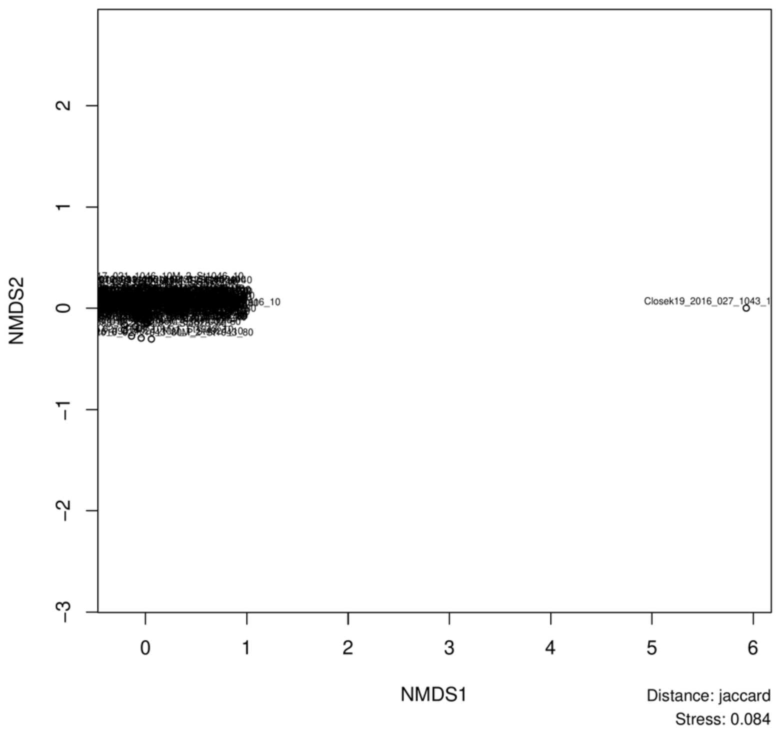

Sampling station

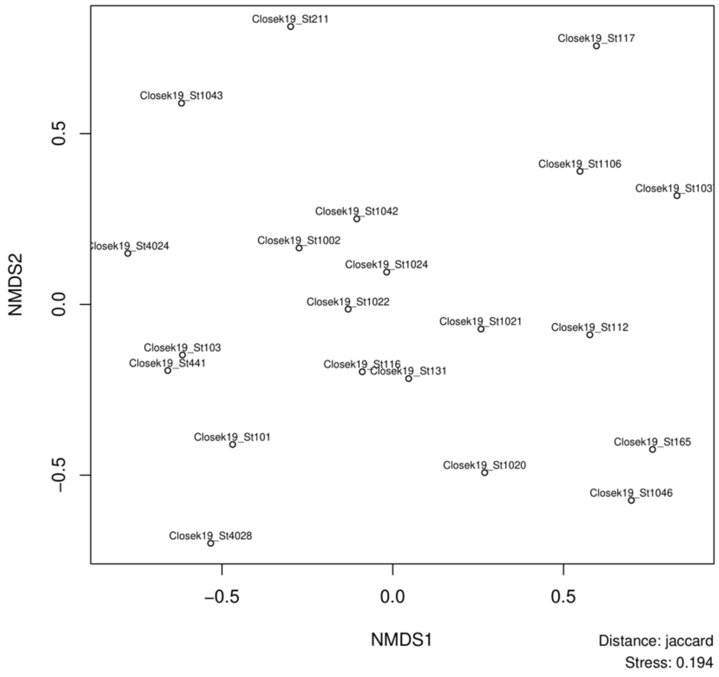

FIGURE S2– 3

hclust

Sampling point

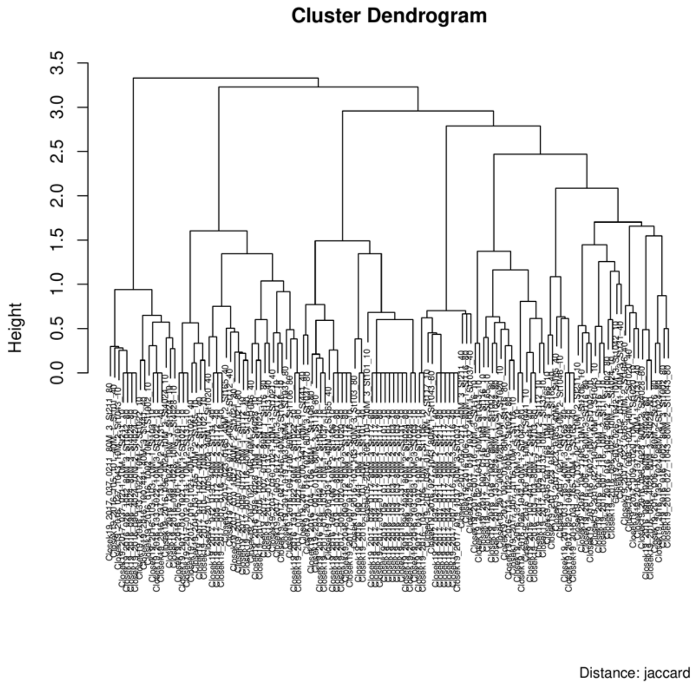

Sampling station

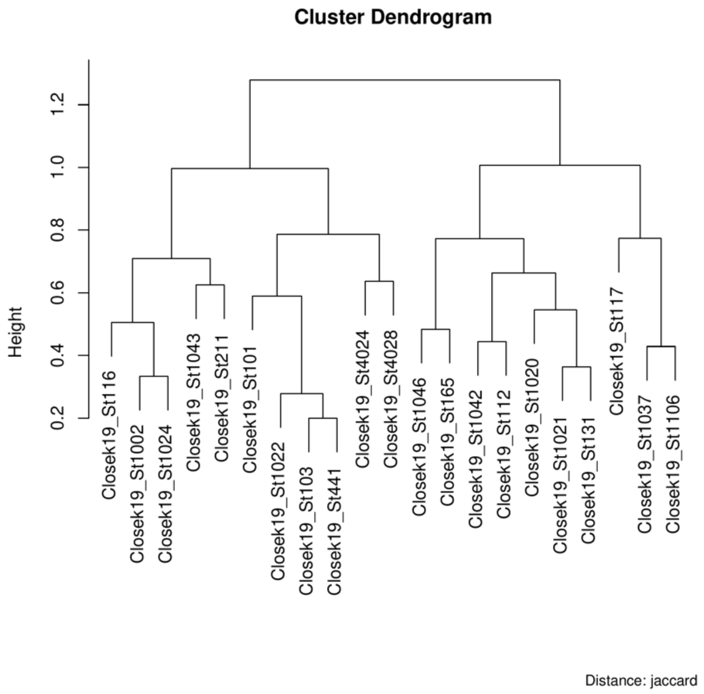

FIGURE S2–4

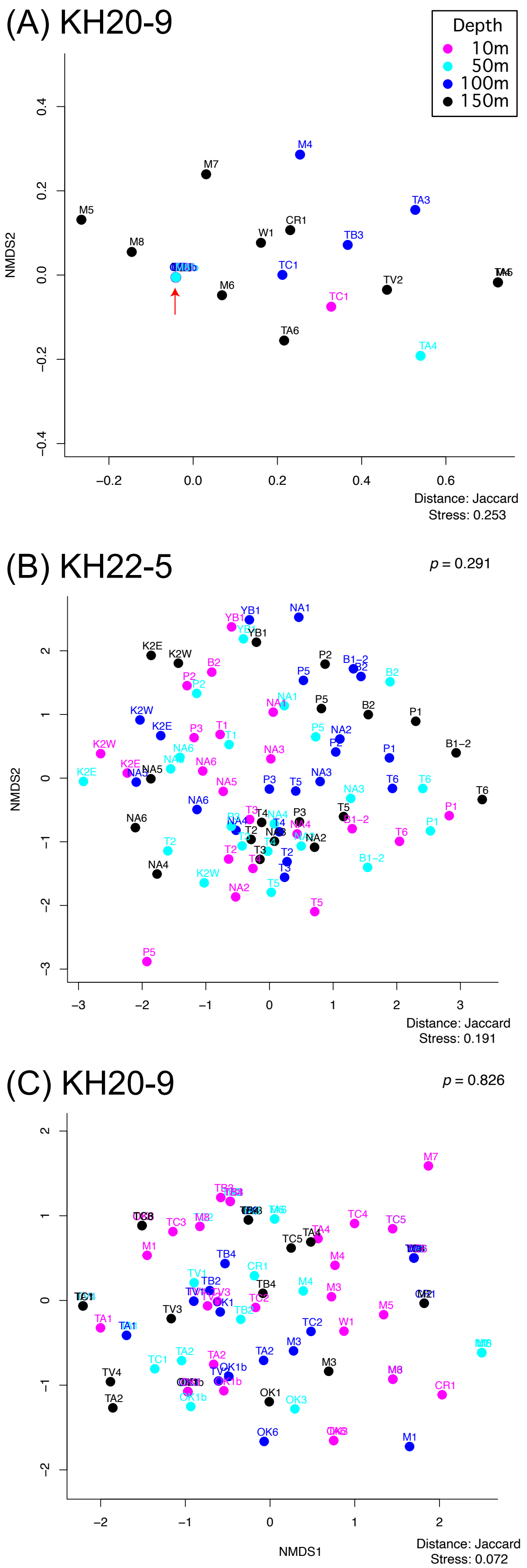
(A) KH20-9
Depth
10m
50m
100m
150m
NMDS2
Distance: Jaccard
Stress: 0.253
(B) KH22-5
p = 0.291
NMDS2
Distance: Jaccard
Stress: 0.191
(C) KH20-9
p = 0.826
NMDS1
Distance: Jaccard
Stress: 0.072

FIGURE S3

(A) KH22-5

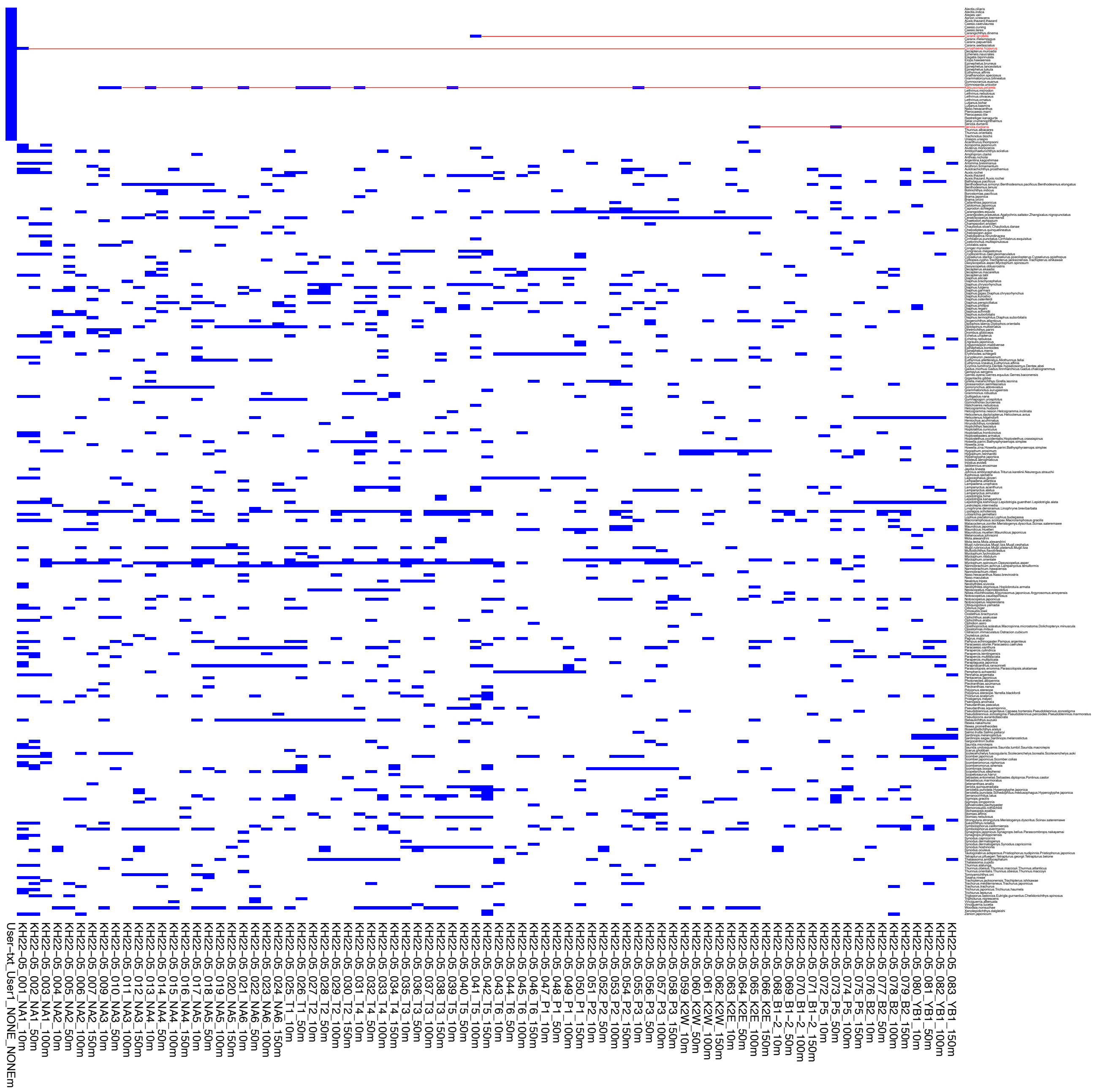

FIGURE S4A

(B) KH20-9

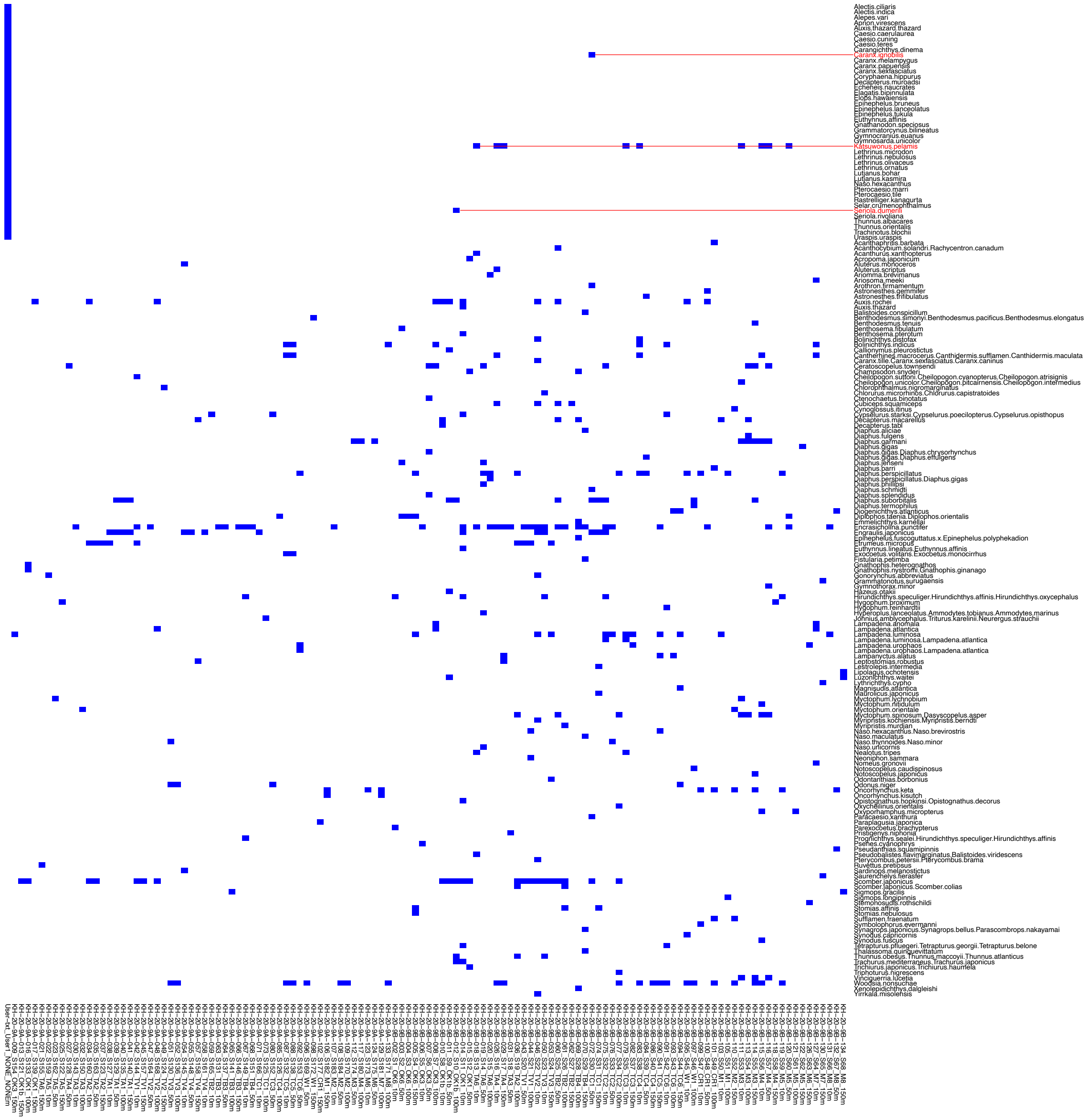

FIGURE S4B

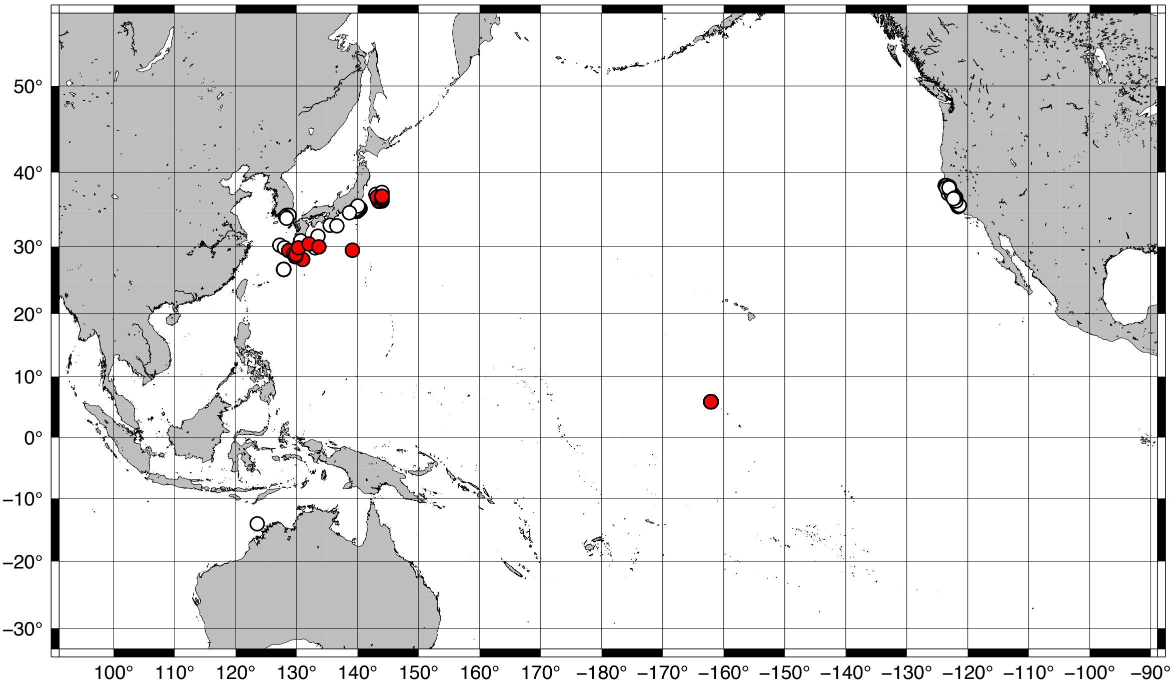

: *Katsuwonus pelamis* detected.

FIGURE S5

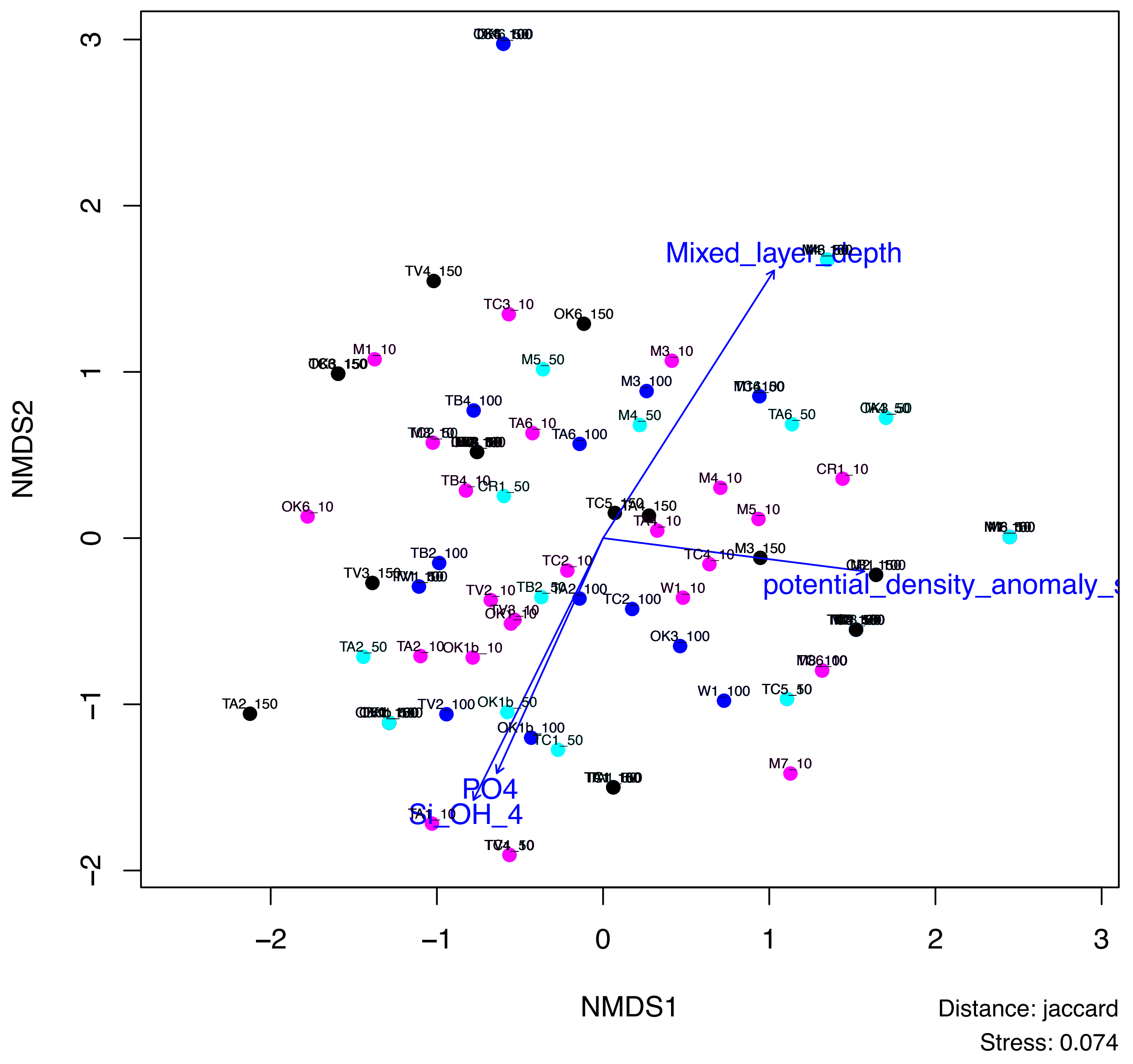

Mixed_layer_depth
potential_density_anomaly_s
PO4
Si_OH_4
NMDS1
NMDS2
Distance: jaccard
Stress: 0.074

FIGURE S6
